# Differential Effects of Decisional and Emotional Forgiveness on Psychological, Spiritual, Social, Volitional, and Physical Well-Being: A Scoping Review

**DOI:** 10.3390/healthcare13090992

**Published:** 2025-04-25

**Authors:** Richard G. Cowden, Everett L. Worthington, Caleb A. Chung, Zhuo Job Chen

**Affiliations:** 1 Human Flourishing Program, Institute for Quantitative Social Science, Harvard University, Cambridge, MA 02138, USA; 2 Department of Epidemiology, Harvard T.H. Chan School of Public Health, Boston, MA 02115, USA; 3 Department of Psychology, Virginia Commonwealth University, Richmond, VA 23284, USA; 4 School of Nursing, University of North Carolina-Charlotte, Charlotte, NC 28223, USA

**Keywords:** decision to forgive, decisional forgiveness, emotional forgiveness, health, scoping review, well-being

## Abstract

Within a stress-and-coping theory of forgiveness, two dimensions of forgiveness have been hypothesized—decisional forgiveness (DF) and emotional forgiveness (EF). Each is theorized to have different impacts on different dimensions of well-being—psychological, spiritual (or religious), social, volitional, and physical. A scoping review was performed to explore the associations of each dimension of forgiveness with each dimension of well-being. A total of *k* = 30 articles met the criteria for inclusion, and estimates of the association between DF and/or EF with one or more indicators of well-being were extracted. Both dimensions of forgiveness were positively linked to all dimensions of well-being, except that there were too few studies on physical well-being (e.g., self-rated physical health) to analyze. DF was generally more strongly related to indicators of spiritual well-being (e.g., faith maturity), psychological well-being (e.g., happiness), and volitional well-being (e.g., conciliatory behavior) than EF, whereas the inverse was observed for social well-being (e.g., marital satisfaction). However, because most studies were cross-sectional, firm conclusions about the associations of both DF and EF with well-being were limited by a paucity of robust studies. Suggestions to guide future research are provided, including the need for more rigorous longitudinal research and better evidence-based theorizing.

## 1. Introduction

The stress-and-coping theory of forgiveness [1] is a widely adopted theory of forgiveness. It also serves as the basis for the REACH Forgiveness intervention [2], which has been shown to be effective in at least 25 randomized controlled trials (RCTs) on psychoeducational group interventions and several on do-it-yourself workbook interventions. The most recent of those was conducted by Ho et al. [3], which enrolled 4598 participants in China, Indonesia, Ukraine (two sites), Colombia, and South Africa to test the effectiveness of a brief do-it-yourself REACH Forgiveness workbook. This was an RCT that had more participants than all previous forgiveness intervention research combined.

The stress-and-coping theory has yielded advances in understanding forgiveness. For example, there are multiple ways of coping with injustices (e.g., pursue justice, see justice enacted, appeal for divine justice), the effects of which may vary. Stressed people might seek to relinquish stressful responses to God. They might also seek to engage in volitional acts that are intended to reduce situational stressors (usually relationship conflict). Such acts might include tolerating injustices (which might reduce situational pressures but usually elevates internal pressures), forbearing (tolerating injustices but done for the good of the relationship or collective), accepting (psychologically distancing or disengaging from the stressful situation to move on with life), or forgiving (an internal, prosocial response that holds perpetrators accountable but may change behavioral intentions, motivations, and emotions toward the offender).

In a broad sense, forgiveness is of four types (i.e., divine forgiveness, self-forgiveness, person-to-person forgiveness, and intergroup forgiveness). The present scoping review focuses on person-to-person forgiveness, a complex whole-person experience that involves reducing negative thoughts, emotions, and behaviors toward a transgressor and replacing them with more positive ones. From a stress-and-coping perspective, there are two dimensions of person-to-person forgiveness [2]. Decisional forgiveness (DF) is a behavioral intention to treat the offender as a valued and valuable person and to foreswear vengeance. Emotional forgiveness (EF) is an emotional replacement of negative unforgiving emotions (e.g., resentment, bitterness, hostility, hatred) with positive other-oriented emotions (e.g., empathy, sympathy, compassion, love). Emotions and motivations are strongly related, so EF (and less certainly DF) can reduce vengeful and (sometimes) avoidant motivations through replacement with benevolent and conciliatory motivations [4]. Emphasizing DF and EF as markers of forgiveness does not imply that the forgiveness process is reducible to these two dimensions.

One measure of EF and two measures of DF have been promulgated. Worthington et al. [5] developed the eight-item Emotional Forgiveness Scale (EFS). Its items seek to operationalize the definition as a replacement of unforgiving emotions with positive other-oriented emotions. They include “I feel sympathy toward him or her” and “I resent what he or she did to me” (reverse scored). Worthington et al. [5] also created the Decisional Forgiveness Scale (DFS) to operationalize the definition of DF. It assesses behavioral intentions and sums them into a total DFS score. For example, its eight items include “I will not seek revenge upon him or her” and “I will not try to help him or her if he or she needs something” (reverse scored). Two distinct, but correlated, four-item subscales were created for both EFS and DFS. For the EFS, the subscales were Presence of Positive Emotions and Reduction of Negative Emotions (correlated at *r* = 0.50 [5]). For the DFS, the subscales were Prosocial Intentions and Inhibition of Harmful Intentions (correlated at *r* = 0.40 [5]). Some empirical studies have used subscale scores instead of (e.g., Holeman et al. [6]) or in addition to total scale scores (e.g., Hook et al. [7]). As an alternative to the DFS, Davis et al. [8] created the Decision to Forgive Scale (DTFS), which seeks to assess a more global conception of a decision to forgive, not tied to its operational definition. This six-item scale assesses a global self-report of whether a person has made a decision to forgive, and if so, to what degree. Its items include “I have decided to forgive him or her” and “My choice is to forgive him or her.”

A reasonable question is whether measures of DF versus EF assess the same or different latent variables. Correlations between measures of DF and EF reported in prior research are generally in the range of *r* = 0.40 to 0.70 (e.g., [9,10]), suggesting that DF and EF are related but distinct constructs. If they are different, might they be related to different outcomes? To consider one of those outcomes, Worthington et al. [11] provided a theoretical introduction to the connections between EF and DF and health. Based on the extant data linking forgiveness to health and well-being, they suggested that EF was related to health. EF corresponds to reduced negative affect of unforgiveness and increased positive affect, suggesting that it might influence psychophysiological changes directly. Worthington et al. [11] suggested that EF would have more direct health and physical well-being consequences than DF, which assessed behavioral intentions towards the offender. They also reviewed peripheral correlates of forgiveness, finding changes in blood pressure, heart rate, and pressure–rate product were correlated to forgiveness (presumably EF, but not explicitly studied as EF). They also found that changes in the central nervous system accompanied decisions about whether an event was forgivable (presumably closer to DF, but not explicitly studied as DF). With that theoretical launching pad, other theoretical differential effects of DF and EF on outcomes are considered below.

### 1.1. Theoretical Differential Effects of Decisional Forgiveness and Emotional Forgiveness on Well-Being Outcomes

Some theorizing within the stress-and-coping theory of forgiveness informs what types of outcomes might respond to DF versus EF. Before considering differences in outcomes generated by DF or EF, it is worthwhile considering the types of well-being outcomes that might be generated. For the purposes of this scoping review, they have been divided into psychological, spiritual, social, volitional, and physical well-being outcomes.

### 1.2. Types of Well-Being Outcomes

Psychological well-being outcomes might involve directly changed cognition, like reduced rumination [12]. They may also include changes in emotion, which might reduce acute experience and expression of anger, anxiety, or sadness [13]. More broadly, they might involve reduced mental health symptoms like depression and anxiety [3] and increased psychological well-being like a sense of peace, calm, lowered arousal, positive attention, openness to experience, and use of positive emotional coping strategies [14]. As time passes, psychological outcomes attending forgiveness might involve reports of posttraumatic growth, resilience, adaptive responses to adversity [15], an expanded sense of self-esteem, enhanced self-compassion, subjective well-being, happiness, sense of meaning and purpose, and life satisfaction.

Spiritual (or religious) well-being outcomes might include reduced religious or spiritual struggles and anger at God [16] and reduced frequency of feelings of sacred loss or desecration [17]. In addition, spiritual outcomes might involve increases in spiritual maturity, religious commitment, or self-rated religiosity or spirituality [18].

Social well-being outcomes might involve general relational ratings of satisfaction with one’s relationship that might have been affected by a transgression [19,20]. Thus, social outcomes might include relational closeness, affection toward the partner, relationship satisfaction or quality, and relationship stability or commitment.

Volitional well-being outcomes might include choices, actions, or experiences that reflect a person’s agency, will, and commitment to live in alignment with their deeply held ethical standards, values, or virtues. Although volitional well-being is increasingly recognized as a crucial dimension of well-being in its own right [21], volitional well-being outcomes are often thought of as intermediate behavioral outcomes that a person might take to seek a higher level of psychological, social, or spiritual outcome. Thus, they can be expected to mediate between forgiveness and other types of well-being. For example, DF or EF might stimulate attempts at self-compassion or efforts at self-regulation, both of which are in turn aimed at better psychological well-being. Similarly, DF or EF might stimulate expressions of forgiveness, which might stimulate in the offending party conciliatory behavior (such as admitting responsibility, confessing wrongdoing, apologizing, expressing empathy for the partner, or offering to make restitution or amends) or expression of gratitude, which in turn might lead to enhanced relational well-being. Alternatively, DF or EF might stimulate prayer, meditation, or the pursuit of other disciplines (e.g., gratitude practices) to support spiritual well-being.

Physical well-being outcomes might involve improvements in health behaviors (e.g., sleeping better, eating healthier) and improvements in physical health conditions. Research on these has accumulated [22].

### 1.3. Differential Hypotheses for Effects of Decisional Forgiveness and Emotional Forgiveness on Well-Being Outcomes

If both DF and EF are effective coping strategies, then psychological variables should be affected by both. However, EF, which replaces negative unforgiving emotions with positive other-oriented emotions, has been hypothesized to more strongly affect psychological outcomes—increasing positive psychological outcomes and decreasing negative ones [23]. Thus, rumination should be expected to succumb to higher EF. On the other hand, DF, which involves changing one’s behavioral intentions, should affect some psychological outcomes, namely negative psychological outcomes, and as emotions change, the drive to ruminate should decrease due to DF as well. DF also should affect relationships if the behavioral intentions are followed through.

Spiritually, DF seems more directly related to reduced appraisals that one has encountered a spiritual loss or desecration [17]. However, EF might play a larger part in religious or spiritual struggles and anger at God [16]. People cannot usually control emotions as well as their behavioral intentions, so a reasonable expectation is that DF would be more strongly related to omnibus religious self-appraisals like spiritual maturity, religious commitment, or self-rated religiosity or spirituality. However, when one does experience EF, it is likely related to other emotion-engaging processes such as reduced religious or spiritual struggles and anger at God.

When a person who has been offended engages in positive volitional behavior, it is likely that their experienced social outcomes will be affected. DF is a behavioral intention to treat the offender differently, as a more valued and valuable person. Thus, DF likely will motivate positive volitional behavior and social outcomes—especially in the early period after an offense. If the victim battles through until they experience EF, then EF, as it manifests and is apprehended by the offender, should also begin to affect relationships. Thus, the time of measurement should be crucial in influencing what kinds of associations are anticipated.

McCullough et al. [24] showed that, in general, forgiveness follows a power curve after an offense. That is, immediate decreases in unforgiveness and increases in forgiveness are experienced. Forgiveness reaches an asymptote after about two to three days. However, such curves homogenize responses over time and generally represent the mean of participant responses. McCullough et al. [24] plotted individual responses and found that forgiveness occurred quickly for many people, dominating the power curve. But substantial numbers of people forgave slowly over time, did not change their level of unforgiveness over time, or even became less forgiving as they ruminated about the event over time. Such patterns need thorough longitudinal research in order to be detected.

Theoretically, EF is more likely than DF to affect physical outcomes [25]. EF involves replacing negative emotions with more positive ones (or at least neutralizing some of the negative emotions), so EF is likely to reduce chronic emotions and, along with them, baseline cortisol, sympathetic nervous system responding, and rumination (which contributes to keeping arousal high). Thus, EF is likely to relate to reduced physical markers of stress. In addition, EF might change negative motivations to more positive or less negative motivations, bringing about less avoidance of the offender and promoting more social and volitional behaviors. Decisions to treat the offender positively might affect health, but the effects are likely less direct than with EF.

### 1.4. The Present Review

This scoping review aims to map the landscape of empirical research that has reported associations of DF and/or EF with well-being outcomes to explore how existing evidence aligns with established theorizing and identify some potential hypotheses for future examination. In particular, this scoping review addresses the following overarching research question: What empirical evidence has been documented about the associations of DF and EF with multidimensional well-being? The exploratory and descriptive synthesis of this scoping review focused principally on studies that have looked at associations for DF or EF with outcomes on at least one of five well-being dimensions (i.e., psychological, spiritual, social, volitional, and physical), which dimensions of well-being received the most attention, and a description of the magnitude of associations observed. Direct associations are emphasized rather than mediation and moderation because a cursory examination of the articles revealed mostly weak methodology (i.e., cross-sectional designs) and few tests of moderation or mediation. Most of the included studies used a cross-sectional design involving contemporaneous assessment of primary study variables. Such studies tend to suffer from interpretive challenges. For example, participants typically rate their responses to transgressions at all durations of time after an offense has occurred. Thus, it is difficult to see time-dependent changes in cross-sectional research results. Moreover, with cross-sectional studies, there is usually no discernible temporal order among the variables, which is necessary for establishing a possible cause-and-effect association. This is important for making decisions about possible targets for intervention. For example, if a negative correlation is observed between EF and contemporaneously assessed depression symptoms, it is not possible to determine which of the two factors should be prioritized when considering options for intervention. Given some of the methodological constraints associated with this area of the empirical literature on forgiveness, the present scoping review does not look microscopically within each dimension of well-being. Rather, the abovementioned theorizing and findings of the scoping review provide a launch platform for exploring novel hypotheses and discussing key areas that need attention in subsequent research.

## 2. Methods

This review was guided by the Joanna Briggs Institute (JBI) methodology for scoping reviews [26]. The central research question was formulated by integrating personal knowledge of the empirical literature on interpersonal forgiveness and well-being among the authors, in conjunction with insights that emerged from preliminary database searches. This scoping review was not preregistered.

### 2.1. Inclusion and Exclusion Criteria

All clinical and nonclinical samples consisting of individuals from any geographic location with any sociodemographic characteristics were included. The phenomena of interest were DF, EF, and psychological, social, spiritual, volitional, or physical well-being. Quantitative studies published in peer-reviewed journals that reported one or more associations linking DF (i.e., DFS or DTFS) and/or EF (i.e., EFS) with one or more indicators of psychological, spiritual (or religious), social, volitional, or physical well-being were included. Qualitative studies, dissertations, conference or meeting abstracts, case studies, commentaries, reviews, and other scholarly articles (e.g., mixed-method studies) and book chapters that did not report any relevant associations were excluded. Eligible publications were restricted to those in English.

### 2.2. Literature Search

The literature search was conducted in three steps, as outlined in the JBI Manual for Evidence Synthesis [26]. Initially, a limited search was performed in PsycINFO to identify potential search terms. These terms were then used to conduct secondary electronic searches in several databases, including CINAHL, PsycINFO, PubMed, and Web of Science. The final searches were conducted on 10 November 2024 and included all available records from inception to that date. The search strategy used in PsycINFO is detailed in Supplemental Table S1, and the format of search expressions was adjusted to fit the requirements of each database.

### 2.3. Screening and Selection

The second step involved importing the retrieved peer-reviewed articles into Covidence and removing duplicates. The third author screened all titles and abstracts for eligibility, while the first author independently screened a random sample of 25%. The third author retrieved the remaining full-text articles, and both the first and third authors independently screened them against the eligibility criteria. Finally, they ensured literature saturation by searching the reference lists of included studies for any additional relevant articles.

**Figure 1:**
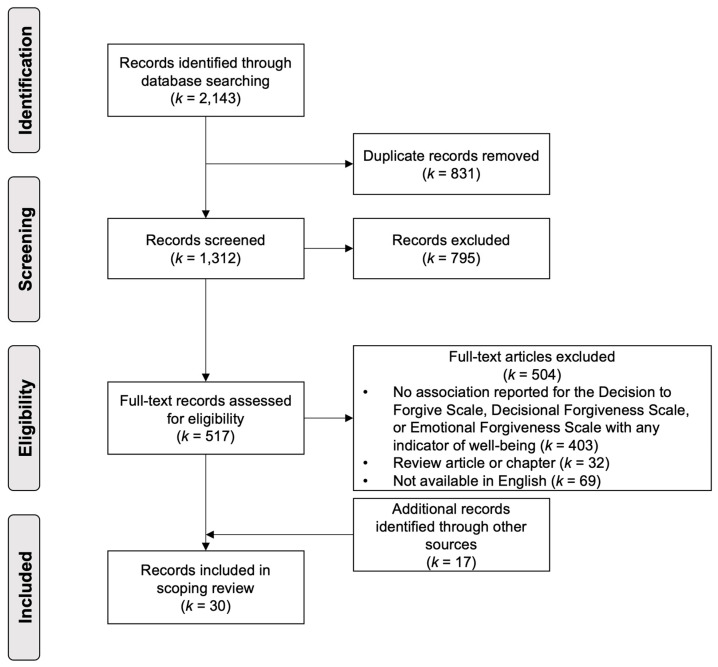
PRISMA-ScR flow chart of the record screening and selection protocol.

A visual display of the study screening and selection process is presented in a Preferred Reporting Items for Systematic Reviews and Meta-Analyses extension for Scoping Reviews (PRISMA-ScR) flow diagram (see Figure 1). After removing duplicates, the electronic database searches yielded a total of 1312 unique records that were potentially relevant. After the title/abstract and full-text screening procedure, 13 records met the criteria for inclusion. A search of references of review articles and chapters that were identified during screening, the references of the records that were eligible for inclusion, and a Google Scholar search for articles with ‘decisional forgiveness’ or ‘emotional forgiveness’ yielded a further collection of 17 records that met the criteria for inclusion. This process yielded a total of 30 published articles for inclusion in the review, all of which are marked with an asterisk (*) in the reference list.

### 2.4. Data Extraction and Synthesis

Relevant data from included studies were extracted and entered into an Excel spreadsheet by the first author. The information that was extracted included publication details (i.e., author/s, year), research methodology (i.e., design, country of data collection, population), participants (i.e., sample size, sex, age, religious affiliation), DF and/or EF measures used, indicator/s of well-being examined in relation to DF and/or EF, and effect sizes corresponding to the associations reported for DF and/or EF with one or more indicators of psychological, spiritual, social, volitional, or physical well-being.

For measures of DF and EF, results were extracted both for total and subscale scores when available. For well-being indicators, results were extracted using total scores of those indicators, unless results for total scores were unavailable (if total and subscale scores were provided, only results for total scores were extracted). Although some measures for indicators of well-being did not align neatly with a single dimension of well-being, each indicator was categorized into one specific dimension. In longitudinal studies, both cross-sectional and prospective associations were extracted when available. Results without covariate adjustment and with covariate adjustment (if they were interpretable) were extracted when available. The extracted data were cross-checked by the third author, and any disagreements were resolved through consultation.

## 3. Results

### 3.1. Study Characteristics

Primary study characteristics are presented in Table 1. Included studies (*k* = 30) were published from 2011 to 2024. Two-thirds (*k* = 20) were published since 2020. Studies contained samples from countries in Europe (*k* = 14; Germany, Poland, Spain), Asia (*k* = 9; China, India, Indonesia, Nepal), North America (*k* = 7; United States), Africa (*k* = 1; South Africa), and Oceania (*k* = 1; New Zealand). Samples from Poland were most common across the included studies (*k* = 11).

Sample sizes ranged from 40 to 1101 (*M* = 328.45). Most studies sampled adults (*k* = 28), usually those in young or middle adulthood. Two studies focused on children or adolescents. Most studies (*k* = 26) included a sample in which 50% or more was female. About one-third of studies (*k* = 9) reported the religious/spiritual affiliations or identities of those who participated (see  Supplemental Table S2). Of those, *k* = 5 studied Christian majority samples.

Most studies (*k* = 22) were cross-sectional in design. Six employed longitudinal designs, and two were interventions. Most articles (*k* = 23) measured both DF and EF. Some (*k* = 6) focused on DF, and one focused on EF. Of the two scales to assess DF, articles were split evenly between DTFS and DFS (*k* = 15 each). As for well-being, the psychological dimension was examined most frequently across the studies (*k* = 17), followed by social (*k* = 11), spiritual (*k* = 9), volitional (*k* = 7), and physical dimensions (*k* = 1). Most studies (*k* = 18) addressed a single dimension of well-being, while the remainder addressed two (*k* = 10), three (*k* = 1), or four dimensions (*k* = 1).

### 3.2. Summary of Findings

A total of 334 effect sizes for associations of DF or EF with one or more indicators of psychological, spiritual, social, volitional, or physical well-being were extracted (for additional details about the specific indicator/s for each dimension of well-being in each study, see Supplemental Table S2). For both DF and EF, each effect size was grouped by subscale of the measure that was used (when applicable and available), whether it was a cross-sectional or longitudinal association, whether adjustment was made for covariates, and the magnitude of the effect size. Magnitude of effect size was classified using Funder and Ozer’s [51] guidelines, which were applied as follows: negligible (*r*/β < 0.05), very small (0.05 ≤ *r*/β < 0.10), small (0.10 ≤ *r*/β < 0.20), medium (0.20 ≤ *r*/β < 0.30), large (0.30 ≤ *r*/β < 0.40), and very large (*r*/β ≥ 0.40). A summary of those findings, broken down by dimension of well-being, is reported in Table 2 (for DF) and Table 3 (for EF). The pattern of findings is explored descriptively rather than meta-analytically, as there was a relatively small number of studies included in the review and most have limited interpretability because they are cross-sectional in design.

### 3.3. Decisional Forgiveness

First, the methods were examined. Of the 145 effect sizes extracted for DF (see Table 2), slightly more than one-third (*m* = 56) involved indicators of psychological well-being. Fewer associations were available for indicators of social (*m* = 34), spiritual (*m* = 32), or volitional well-being (*m* = 20); few associations were found for indicators of physical well-being (*m* = 3). Almost two-thirds of all associations were cross-sectional without covariate control (*m* = 85), although some (*m* = 22) cross-sectional associations did adjust for covariates. Fewer than one-third of all associations were longitudinal (*m* = 38), and less than half of those adjusted for potential confounders (*m* = 18).

Second, the content was examined. Almost all non-negligible associations were in the expected direction (i.e., higher values of DF were related to better well-being). However, seven associations (mostly very small/small in magnitude) were not in the expected direction (five of which were cross-sectional), such that DF was related to lower well-being. For the non-negligible associations that were in the expected direction, the effect sizes for each dimension of well-being were varied. Looking at the zero-order cross-sectional associations for DF, the column that had the greatest number of effect sizes with indicators of well-being, the mode/median, respectively, for the magnitude of associations found linking DF with better well-being for each dimension were medium (mode)/medium (median) in size for psychological well-being; small-large (tie)/medium for social well-being; large/small for spiritual well-being; and large/large for volitional well-being. With so few effect sizes for physical well-being (*m* = 3), any summary is limited in generalizability. It is important to note that most dimensions had very few effect sizes, which suggests a reason that the mode and median differed for some dimensions of well-being.

### 3.4. Emotional Forgiveness

Methodologically, of the 189 effect sizes extracted for EF (see Table 3), almost half (*m* = 84) involved indicators of psychological well-being. Fewer associations were available for indicators of social (*m* = 44), spiritual (*m* = 33), or volitional well-being (*m* = 25); few associations were found for indicators of physical well-being (*m* = 3). Almost two-thirds of all associations were cross-sectional without covariate control (*m* = 120), although there were some cross-sectional associations that adjusted for covariates (*m* = 20). Fewer than one-third of all associations were longitudinal (*m* = 49), and about a third of those adjusted for potential confounders (*m* = 17). These findings mirrored the methodological findings for DF.

As for content, almost all non-negligible associations were in the expected direction (i.e., higher values of EF related to better well-being). However, six associations (mostly very small/small in magnitude) were not in the expected direction (five of which were cross-sectional), such that EF was related to lower well-being. For the non-negligible associations that were in the expected direction, the effect sizes for each dimension of well-being were varied. Looking at the zero-order cross-sectional associations for EF, the column that had the greatest number of effect sizes with indicators of well-being, the mode/median, respectively, for the magnitude of associations found linking EF with better well-being for each dimension of well-being were small/small for psychological well-being; very large/large for social well-being; very small-small (tie)/very small for spiritual well-being; and small-very large (tie)/medium for volitional well-being. Once again, it is important to note that most dimensions had very few effect sizes, which suggests a reason that the mode and median differed for some dimensions of well-being.

## 4. Discussion

This scoping review addressed the following overarching research question: What empirical evidence has been documented about the associations of DF and EF with multidimensional well-being? This question was explored by examining the effects of DF and EF on five dimensions of well-being. After reflecting on the methods and the findings of the studies reviewed, methodological, theoretical, and empirical suggestions about the way forward are discussed.

### 4.1. Methodology

The scientific study of DF and EF in relation to well-being, in general, is still dominated by cross-sectional correlational research, although some longitudinal and intervention research has been conducted. It is clear from Table 2 and Table 3 that when systematic covariates are considered in observational studies, which is more possible with longitudinal designs and when time lags are considered, effect sizes tend to be somewhat weaker. Accounting for a few covariates in cross-sectional designs does not appear to be particularly informative, especially when compared to longitudinal designs that control for a greater number of potential confounders. Even when high-quality longitudinal designs and more sophisticated analytical methods were used, effects relating DF and EF to the various aspects of well-being were mostly still present. Nevertheless, it is a word to the wise that future observational research needs to emphasize the use of longitudinal designs to discover what the true relationships are among the variables considered in this scoping review. Given that research reporting on associations of DF and EF with indicators of well-being now spans more than a decade, it is time that observational research in this area moves beyond single-shot cross-sectional studies and methodological rigor is given priority (at least on par with, if not of greater priority than, novelty). Empirical evidence in this area of the literature on forgiveness will move forward at an incrementally slow pace if research gaps are filled with observational studies that are methodologically weak. Although this call is principally for empirical researchers studying forgiveness, it is a call that needs to be supported by relevant funders as well as journal editors and reviewers.

### 4.2. Content

Based on the current evidence available, this scoping review has limited power or ability to compare differences in how DF versus EF relate to aspects of well-being. Despite the limits of this scoping review, the findings point to some possible ways in which theorizing using DF and EF might need refinement. Perhaps the strongest hypothesis about forgiveness and well-being is that EF would be especially strongly related to lower psychological distress and higher psychological well-being [2,23]. The correlational data tell a different story. For EF, both mode and median effect sizes were small, contrary to hypothesized relationships. For DF, however, medium effect sizes for both mode and median exist between DF and psychological well-being. One possible explanation for these unanticipated results might be that seeing one’s own forgiving behavior, which is posited if forgivers actually follow through on the behavioral intentions of DF, might be stronger than emotional feelings of forgiveness. This is consistent with Bem’s [52] self-perception theory, in which seeing one’s own behavior can overshadow one’s internal feelings. Obviously, the limitations of correlational research might influence the findings. But, this counter-theoretical finding—even with weak methodology—should give theoreticians pause to reconsider.

Interpersonal relations are affected by both DF and EF. DF is defined as changes in behavioral intention toward an offender that eschew revenge and seek to treat the offender as a valued and valuable person [2]. Thus, theory suggests that DF affects relationships more than EF, especially in the early hours or even days after experiencing DF. Later, once EF’s effects have trickled into relationship behavior, EF might be expected to make more of an impact. Using correlational studies, because these studies are sufficiently numerous to draw any conclusions, Table 2 and Table 3 indicate that social well-being has a modal relationship with DF that is small/large and a median relationship that is medium in effect size. On the other hand, EF has a modal relationship with social well-being that is very large and a median relationship that is large in effect size. Although these findings are unexpected, there may be some potential explanations. First, the designs are correlational and do not consider time course, homogenizing the correlations when measures are not separated by time. Second, it is possible that EF is simply a stronger determinant of social well-being. More longitudinal research is needed to untangle this time-dependent hypothesis.

Another strong and well-supported hypothesis is the effect of DF on spiritual well-being [18]. The effect of DF is hypothesized to be stronger than the effect of EF on spiritual well-being. This was based on theorizing from Christian theology, which prioritizes making a decision to forgive someone in obedience to God, although the theorizing could be extended to other religions [53]. Making behavioral intention statements (i.e., DF) is thought to be under more volitional control than controlling one’s emotions. Theologies from other religions and philosophies that are non-theistic (like Buddhism) are needed to flesh out this hypothesis. However, this hypothesis is supported by the cross-sectional data. The magnitude of effects for DF using the mode of cross-sectional studies is large, and using the median is small. For EF, the mode effect size is very small/small in magnitude, and the median is very small. Thus, DF appears to be more strongly related to spiritual well-being than EF, which supports prior theorizing.

This scoping review touches on a previously unarticulated hypothesis—that volitional well-being outcomes are typically mediational outcomes (often choices or actions) that lead to better psychological, social, or spiritual well-being. However, volitional well-being is important to look at in and of itself as a key dimension of well-being [21]. Prior theorizing in the scientific literature on forgiveness has not focused on volitional well-being outcomes per se. However, if volitional well-being outcomes are theorized as typically involving choices or actions, it is reasonable to assume that DF will influence them more than EF. This speculation is borne out by the cross-sectional results. The connections between DF and volitional well-being outcomes were large in magnitude for both mode and median. The connections between EF and volitional well-being outcomes were small/very large in magnitude for the mode and medium for the median.

The results of this scoping review did not provide an opportunity for appropriate commentary about the potential effects of DF and/or EF on physical well-being because there were too few effect sizes. In a prior meta-analysis, Lee and Enright [22] examined 128 studies (*N* = 58,531) relating forgiveness to physical health. The mean effect size was *r* = 0.14. No moderators affected the relationship. Clearly, associations of forgiveness with health behaviors and outcomes have been investigated often, but not often using explicit measures of DF and EF, which accounts for the few studies that met the criteria for inclusion in this scoping review.

### 4.3. Future Directions

#### 4.3.1. Methodology

Four methodological recommendations are proposed. First, meta-analyses are needed. The literature is accruing to the point where, within the next few years (if sufficiently rigorous studies are performed), a high-quality meta-analysis would be called for to address differential effects of DF and EF on dimensions of well-being. Various moderators could be investigated if 10 to 20 additional rigorous studies could be conducted.

Second, future studies are needed to investigate potential differences between the DFS and the DTFS. The content assessed by these measures is different, even though the relationships with well-being outcome variables are similar. However, additional studies and a meta-analysis might be needed to unravel the subtleties of what the two measures are assessing.

Third, the preponderance of observational studies using cross-sectional designs aligns with calls for more longitudinal designs [54]. It is important to emphasize that the present discussion in this scoping review mostly relied on cross-sectional studies for theorizing, and even cross-sectional studies that had no variance from covariates removed. Longitudinal designs can investigate potential causal relationships—at least within a sample. They can (often) also assess more covariates simultaneously. Prospective studies are also particularly needed. In the present scoping review, longitudinal studies available for consideration were not very informative for drawing conclusions. One reason is that they comprised about one-fifth of the studies. Thus, they were not good candidates from which to draw firm conclusions. Longitudinal research with many participants, allowing numerous covariates to be evaluated, can be most helpful in determining the true strength of relationships between DF or EF and indicators of well-being.

Consider one recent longitudinal study where Cook et al. [25] surveyed 595 primarily young adult Indonesians. The intent was to investigate the independent contributions of DF and EF to ameliorating different types of distress and also to improving various aspects of well-being. The authors used a three-wave longitudinal design over three contiguous months from the end of 2021 to the beginning of 2022 and applied an outcome-wide analytic template to analyze the data. They computed the effects of DF and EF with three indicators of distress and with ten aspects of well-being, adjusting for numerous covariates, including pre-baseline values of DF and EF and the 13 outcomes. DF assessed at T2 was associated with increases at T3 in life satisfaction, self-rated physical health, sense of purpose, an orientation to promote good, delayed gratification, contentment in relationships, and satisfaction with relationships. EF assessed at T2 was associated with an increase at T3 in satisfaction with relationships. Neither DF nor EF at T2 was associated with any of the subsequent indicators of distress assessed at T3. Cook et al.’s findings suggest that DF might have more short-term benefits on well-being than EF. Potentially, such findings might be due to (1) EF taking longer to develop than DF; (2) Indonesia being a collectivistic country, which has been hypothesized to be more attuned to DF than EF (see Kurniati et al. [29]); or (3) assessing other dimensions of well-being (e.g., psychological) more than social well-being.

#### 4.3.2. Content

In this scoping review, the results from the included studies often prompted a pause in considering extant theorizing at face value. Importantly, both DF and EF generally showed positive relationships with aspects of well-being. Forgiveness is good for the forgiver. Beyond that generalization, DF looked like it might be a more powerful predictor of psychological, spiritual, and volitional well-being than EF. On the other hand, EF seemed to be a better predictor of social well-being than DF. These tentative, empirically based (rather than conceptually based) hypotheses need empirical replication and theoretical justification.

The present scoping review highlighted a potential expansion in theorizing involving volitional well-being outcomes, which were found to be related more strongly to DF than EF. This might be due to the cross-sectional nature of most of the studies that were included in this review. At any rate, it was posited that volitional choices or actions affected directly by DF (especially) and EF might serve as mediators between DF and EF and psychological, social, and spiritual well-being outcomes. However, longitudinal or experimental research is needed to investigate mediation.

Finally, the studies relating DF and EF to physical health behaviors or outcomes were embarrassingly few in number. Thus, future research must investigate physical health benefits more frequently. Knowing whether DF or EF more strongly contributes to different outcomes seems important—too important to rely solely on generic measures of “forgiveness.”

## 5. Conclusions

This scoping review pointed to several future directions in two areas. In methodology, more sophisticated longitudinal research is needed. In content, the findings questioned theorizing that has heretofore suggested DF might be most closely related to social and spiritual well-being and EF might be most closely related to psychological and physical well-being. The findings of this scoping review showed—very tentatively based mostly on correlational research—that DF indeed seems more strongly related to spiritual well-being. But, contrary to theorizing, EF seems more strongly related to social well-being, while DF seems more strongly related to psychological well-being.

## Figures and Tables

**Table 1:** Summary of studies reporting associations of decisional and/or emotional forgiveness with indicator/s of well-being.

Publication Details		Research Methodology				Participants			
**Author/s**	**Year**	**Design**	**Forgiveness Measure/s**	**Dimension/s of Well-Being Included ^a^**	**Study Population**	**Resident** **Country ^b^**	**Sample Size ^c^**	**Sex**	**Age (yrs)**
Holeman et al. [6]	2011	CS	DFS EFS	PS (1 indicator) SP (3 indicators)	University students	US	437	♀ (64%)	*M* = 31.9 ± 13.5
Watkins et al. [27]	2011	CS	DFS (3-item PI) EFS (PPE)	V (1 indicator)	University students	NP	221	♀ (40%)	≤25 (84%) 26+ (16%)
Scherer et al. [28]	2012	CS	DFS EFS	V (1 indicator)	University students hurt by immediate family member due to their alcohol misuse	US	141	♀ (67%)	*M* = 19.3 ± 1.9 (range: 18–29)
Hook et al. [7]	2013	CS	DFS (7 items) EFS	SO (1 indicator) V (1 indicator)	University students	CN NZ	CN: 172 NZ: 91	CN: ♀ (38%) NZ: ♀ (63%)	CN: - 25 (26%) - 21–25 (72%) - 26+ (2%) NZ: - 25 (70%) - 21–25 (21%) - 26+ (9%)
Davis et al. [8] (Study 3)	2015	CS	DTFS	SP (1 indicator)	University students who experienced major hurt by a religious/spiritual leader	US	335	♀ (61%)	*M* = 24.8 ± 6.8
Kurniati et al. [29] (Study 2)	2017	CS	DFS EFS	PS (1 indicator)	Adolescents in secondary school	ID	424	NR	*M* = 15.5
Chi et al. [30]	2019	CS	DFS EFS	SO (3 indicators)	Adults who had experienced spousal infidelity	CN	154	♀ (81%)	*M* = 36.1 ± 6 (range: 26–56)
Cowden et al. [31]	2019	CS	DFS EFS	PS (3 indicators)	Adult females in a heterosexual relationship	ZA	515	♀ (100%)	*M* = 29.5 ± 10.1 (range: 18–77)
Choe and Davis [32]	2020	CS	DTFS	SP (1 indicator)	University students	US	238	♀ (75%)	Range: 19–58
Hong et al. [33]	2020	CS	DFS EFS	PS (1 indicator)	Primary and secondary school students (4th to 8th grade)	CN	1009	♀ (47%)	*M* = 11.8 ± 1.3 (range: 10–15)
Major et al. [34]	2020	CS	DFS (7 items)	PS (1 indicator)	University students	US	292	♀ (55%)	*M* = 19.2 ± 1.4 (range: 18–28)
Kaleta and Mróz [35] (Study 2)	2021	CS	DTFS EFS	PS (1 indicator)	General adult population	PL	236	♀ (73%)	*M* = 23.9 ± 7.6 (range: 15–75)
Byra et al. [9]	2022	CS	DTFS EFS	PS (1 indicator) SO (1 indicator)	Adults with physical disabilities	PL	267	♀ (46%)	*M* = 40.5 ± 11.6 (range: 17–69)
Mróz and Kaleta [36]	2022	CS	DTFS	PS (1 indicator)	Adults who have experienced cyberhate	PL	246	♀ (73%)	*M* = 23.7 ± 7 (range: 18–50)
Mróz et al. [37]	2022	CS	DTFS EFS	PS (4 indicators)	General adult population	PL	650	♀ (76%)	*M* = 27.9 ± 11.1 (range: 18–79)
Wang et al. [38] (Study 2)	2022	CS	DFS EFS	PS (2 indicators)	University students	CN	1101	♀ (64%)	*M* = 22.6 ± 3.5
Wu et al. [39]	2022	CS	DFS EFS (7 items)	SO (1 indicator) V (1 indicator)	University students currently in a romantic relationship	CN	103	♀ (62%)	*M* = 19.4 ± 1.3 (range: 18–23)
Mróz et al. [40]	2023	CS	DTFS EFS	SP (1 indicator)	General adult population	PL	396	♀ (69%)	*M* = 28.9 ± 12.3 (range: 18–79)
Byra [41]	2024	CS	DTFS EFS	PS (1 indicator) SO (1 indicator)	Mothers of children with disabilities	PL	174	♀ (100%)	*M* = 34.1 ± 11
Kaleta et al. [42]	2024	CS	DTFS EFS	SO (1 indicator)	Adult amputees	PL	102	♀ (57%)	*M* = 41.7 ± 14.4 (range: 18–75)
Mróz et al. [43]	2024	CS	DTFS EFS	PS (1 indicator) SP (1 indicator)	Adults who had experienced childhood abuse from a close family member	PL	309	♀ (64%)	*M* = 30.9 ± 11.9 (range: 18–68)
Skalski-Bednarz et al. [44]	2024	CS	DTFS	PS (1 indicator) SP (3 indicators)	Adult Ukraine war refugees	DE PL	243	♀ (82%)	*M* = 37.6 ± 8.9 (range: 18–68)
He et al. [45]	2018	L ^d^	DFS EFS	SO (2 indicators)	Married couples	CN	268 couples	♀ (50%)	♂: *M* = 29.6 ± 3.3 ♀: *M* = 28.1 ± 2.5
Gámiz et al. [46]	2021	L ^d^	DTFS	SO (1 indicator)	University students	DE ES	191	♀ (72%)	*M* = 23.9 ± 9.5
Cook et al. [25]	2022	L ^d^	DTFS EFS	PH (1 indicator) PS (8 indicators) SO (2 indicators) V (2 indicators)	University students and the general adult population	ID	595	♀ (55%)	*M* = 22 ± 4.4 (range: 18–55)
Cornish et al. [47]	2024	L ^d^	EFS	PS (7 indicators) SO (3 indicators) V (3 indicators)	Adults in a romantic relationship for ≥ 1 year	US	302	♀ (59%)	NR
Skalski-Bednarz and Toussaint [48]	2024	L ^d^	DTFS EFS	PS (1 indicator) SP (1 indicator)	Adult female sexual assault survivors	PL	246	♀ (100%)	*M* = 29 ± 7.3 (range: 18–45)
Skalski-Bednarz et al. [49]	2024	L ^d^	DTFS EFS	SP (1 indicator)	General adult population	PL	292	♀ (64%)	*M* = 40.6 ± 13.5 (range: 18–65)
Sandage et al. [10]	2015	I	DFS EFS	PS (1 indicator) SO (1 indicator)	Treatment-seeking adults meeting criteria for borderline personality disorder	US	40	♀ (88%)	*M* = 40 ± 12.9 (range: 19–63)
Toussaint et al. [50]	2020	I	DFS EFS	SP (3 indicators) V (1 indicator)	University students	IN	124	♀ (56%)	*M* = 23 ± 1.4 (range: 21–30)

Note. CS = Cross-sectional, L = Longitudinal, I = Intervention, DF = Decisional forgiveness, DFS = Decisional Forgiveness Scale, DTFS = Decision to Forgive Scale, EF = Emotional forgiveness, EFS = Emotional Forgiveness Scale, PI = Prosocial Intention subscale, PPE = Presence of Positive Emotions subscale, NR = Not reported. ^a^ Details about specific indicator/s assessed for the dimension/s of well-being in each study can be found in Supplemental Table S2. Abbreviations for dimensions of well-being are as follows: PH = Physical, PS = Psychological, SO = Social, SP = Spiritual, V = Volitional. ^b^ Country abbreviations are as follows: CN = China, DE = Germany, ES = Spain, ID = Indonesia, IN = India, NP = Nepal, NZ = New Zealand, PL = Poland, US = United States, ZA = South Africa. ^c^ Analytic sample size may differ from total reported sample size. ^d^ Additional details for longitudinal designs are as follows: He et al. [45] (3 waves: 12-month lag), Gámiz et al. [46] (5 waves: Wave 1 followed by 2, 5, 8, and 12 days after Wave 1), Cook et al. [25] (3 waves: 1-month lag), Cornish et al. [47] (3 waves: 1-week lag), Skalski-Bednarz and Toussaint [48] (2 waves: 8-month lag), Skalski-Bednarz et al. [49] (2 waves: 6-month lag).

**Table 2:** Effect size magnitudes for studies reporting associations of decisional forgiveness with indicators of well-being summarized by dimension of well-being.

Magnitude of Association	DF (Total Score)				IHI		PI		Grand Total Number of Effect Sizes
	CS		L		CS		CS		
	ZO	C	ZO	C	ZO	C	ZO	C	
**Psychological well-being** ^a^									
Negligible				4					4
Very small	4 (1)	2		2	1				9 (1)
Small	7	(1)	5	2	1		2		17 (1)
Medium	12	1	4						17
Large	3	2							5
Very large	2								2
Total number of effect sizes	28 (1)	5 (1)	9	8	2	0	2	0	54 (2)
**Social well-being** ^b^									
Negligible				1					1
Very small	1	2	2						5
Small	5 (1)	1	1	1	1				9 (1)
Medium	4		3	1	1		2		11
Large	5								5
Very large	2								2
Total number of effect sizes	17 (1)	3	6	3	2	0	2	0	33 (1)
**Spiritual well-being** ^c^									
Negligible	1				2	1	1	1	6
Very small	2	1 (1)				(1)			3 (2)
Small	3			1	1		2	1	8
Medium	2	2		1 (1)					5 (1)
Large	4	1	2						7
Very large									0
Total number of effect sizes	12	4 (1)	2	2 (1)	3	1 (1)	3	2	29 (3)
**Volitional well-being** ^d^									
Negligible		1							1
Very small									0
Small	1			2 (1)	1				4 (1)
Medium	1	2	2						5
Large	4	1			1				6
Very large	1						2		3
Total number of effect sizes	7	4	2	2 (1)	2	0	2	0	19 (1)
**Physical well-being** ^e^									
Negligible									0
Very small									0
Small	1			1					2
Medium			1						1
Large									0
Very large									0
Total number of effect sizes	1	0	1	1	0	0	0	0	3
Grand total number of effect sizes	65 (2)	16 (2)	20	16 (2)	9	1 (1)	9	2	138 (7)

Note. C = Covariate adjusted associations, CS = Cross-sectional, DF = Decisional forgiveness, IHI = Inhibition of harmful intentions, L = Longitudinal, PI = Prosocial intentions, ZO = Zero-order associations. Non-parenthetical values indicate the number of effect sizes that suggested forgiveness scores were associated with better well-being, with the exception of values in the “negligible” rows, where the direction of association was not closely examined because the reported effect sizes were close to zero. Values in parentheses indicate the number of effect sizes that suggested forgiveness scores were related to lower well-being. Magnitudes for unadjusted (zero-order correlations) associations are as follows: negligible (*r* < 0.05), very small (0.05 ≤ *r* < 0.10), small (0.10 ≤ *r* < 0.20), medium (0.20 ≤ *r* < 0.30), large (0.30 ≤ *r* < 0.40), very large (*r* ≥ 0.40). Magnitudes for covariate-adjusted associations are as follows: negligible (β < 0.05), very small (0.05 ≤ β < 0.10), small (0.10 ≤ β < 0.20), medium (0.20 ≤ β < 0.30), large (0.30 ≤ β < 0.40), very large (β ≥ 0.40). ^a^ Psychological well-being in [6,9,10,25,29,31,33,34,35,36,37,38,41,43,44,48]. ^b^ Social well-being in [7,9,10,25,30,39,41,42,45,46]. ^c^ Spiritual well-being in [6,8,32,40,43,44,48,49,50]. ^d^ Volitional well-being in [7,25,27,28,39,50]. ^e^ Physical well-being in [25].

**Table 3:** Effect size magnitudes for studies reporting associations of emotional forgiveness with indicators of well-being summarized by dimension of well-being.

Magnitude of Association	EF (Total Score)				RNE			PPE			Grand Total Number of Effect Sizes
	CS		L		CS		L	CS		L	
	ZO	C	ZO	C	ZO	C	ZO	ZO	C	ZO	
**Psychological well-being** ^a^											
Negligible	4			6				5			15
Very small	2			2	2			3 (2)		1	10 (2)
Small	7	1	9		6	1		2		1	27
Medium	3	1			3	1	1	2	1	1	13
Large	5				2		2		(1)		9 (1)
Very large	3	1			2			1			7
Total number of effect sizes	24	3	9	8	15	2	3	13 (2)	1 (1)	3	81 (3)
**Social well-being** ^b^											
Negligible					1						1
Very small	(1)			2	1 (1)	1					4 (2)
Small	4		2		2			2			10
Medium	3	1	2					1			7
Large	3		1					1	1		6
Very large	5	1	1				3	1		3	14
Total number of effect sizes	15 (1)	2	6	2	4 (1)	1	3	5	1	3	42 (2)
**Spiritual well-being** ^c^											33
Negligible	1				1			3	1		6
Very small	2			(1)	1			2			5 (1)
Small	2			2	3	3		3	2		15
Medium			1		3						4
Large			1								1
Very large	1										1
Total number of effect sizes	6	0	2	2 (1)	8	3	0	8	3	0	32 (1)
**Volitional well-being** ^d^											25
Negligible				2							2
Very small			2								2
Small	2	1		1					1		5
Medium	1				3			1			5
Large	1				2			2			5
Very large	2	1						3			6
Total number of effect sizes	6	2	2	3	5	0	0	6	1	0	25
**Physical well-being** ^e^											3
Negligible				1							1
Very small											0
Small	1		1								2
Medium											0
Large											0
Very large											0
Total number of effect sizes	1	0	1	1	0	0	0	0	0	0	3
Grand total number of effect sizes	52 (1)	7	20	16 (1)	32 (1)	6	6	32 (2)	6 (1)	6	183 (6)

Note. C = Covariate adjusted associations, CS = Cross-sectional, EF = Emotional forgiveness, L = Longitudinal, PPE = Presence of positive emotions, RNE = Reduction of negative emotions, ZO = Zero-order associations. Non-parenthetical values indicate the number of effect sizes that suggested forgiveness scores were associated with better well-being, with the exception of values in the “negligible” rows where the direction of association was not closely examined because the reported effect sizes were close to zero. Values in parentheses indicate the number of effect sizes that suggested forgiveness scores were related to lower well-being. Magnitudes for unadjusted (zero-order correlations) associations are as follows: negligible (*r* < 0.05), very small (0.05 ≤ *r* < 0.10), small (0.10 ≤ *r* < 0.20), medium (0.20 ≤ *r* < 0.30), large (0.30 ≤ *r* < 0.40), very large (*r* ≥ 0.40). Magnitudes for covariate-adjusted associations are as follows: negligible (β < 0.05), very small (0.05 ≤ β < 0.10), small (0.10 ≤ β < 0.20), medium (0.20 ≤ β < 0.30), large (0.30 ≤ β < 0.40), very large (β ≥ 0.40). ^a^ Psychological well-being in [6,9,10,25,29,31,33,35,37,38,41,43,47,48]. ^b^ Social well-being in [7,9,10,25,30,39,41,42,45,47]. ^c^ Spiritual well-being in [6,40,43,48,49,50]. ^d^ Volitional well-being in [7,25,27,28,39,47,50]. ^e^ Physical well-being in [25].

## Data Availability

Not applicable.

## Supplementary Materials

The following supporting information can be downloaded at: https://www.mdpi.com/article/10.3390/healthcare13090992/s1, Table S1: Search Strategy used in PsycINFO; Table S2: Additional Study Characteristics Details for Studies Reporting Associations of Decisional and/or Emotional Forgiveness with Indicator/s of Well-being.
