# Supplementary material for: Differential Effects of Decisional and Emotional Forgiveness on Psychological, Spiritual, Social, Volitional, and Physical Well-Being: A Scoping Review
Source: Healthcare (Basel). 2025 Apr 25;13(9):992. doi: 10.3390/healthcare13090992 (PMC12071234; doi:10.3390/healthcare13090992)
Supplement: Supplementary file 1 [file healthcare-13-00992-s001.zip › healthcare-3472023-supplementary.pdf]

Differential effects of decisional and emotional forgiveness on psychological, spiritual, social, volitional, and physical well-being: A scoping review

SUPPLEMENTAL FILE

Supplemental Table S1

Search Strategy used in PsycINFO

TI forgiv* AND TI (Happiness OR Happy OR (Positiv* AND (affect* OR emotion)) OR Grief OR grieving OR Bereave* OR Sad OR sadness OR Joy OR joyous OR joyful OR Peace OR Pleasure OR hope OR hopeful* OR (Satisf* AND life) OR Subjective well-being OR Quality-of-life OR Dissatisf* OR Distress* OR ((emotion* OR Mental) AND (trauma OR stress OR illness OR disorder OR health OR Well-being OR well being OR wellbeing)) OR Internalizing OR Depressi* OR Anxiet* OR Trauma OR Stress OR Posttraumatic OR post-traumatic OR (psychiatric AND (disorder OR illness OR symptom*)) OR (Psychologic* AND (Well-being OR well being OR wellbeing OR trauma* OR function* OR adjust* OR distress* OR stress)) OR (Health-related AND quality-of-life) OR Health status OR Health* OR Sick OR sickness OR ((Physical OR Chronic OR terminal) AND (whole* OR function* OR illness* OR disease*)) OR Pain OR Physical disorder OR Symptom* OR Meaning* OR Transcenden* OR Justice OR Courage OR Fortitude OR Wisdom OR Prudence OR Temperance OR Self-control OR Self-regulation OR Moral* OR Ethic* OR Patience OR Gratitude OR Humility OR Hope OR Charity OR Love OR Compassion* OR Altruis* OR Moderation OR ((Post-traumatic OR posttraumatic OR personal) AND growth) OR Lonel* OR Belonging* OR (Social AND (function* OR relation* OR network* OR capital OR support OR connect* OR inclusion OR exclusion OR isolation)) OR (Relation* AND (Qualit* OR satisfy*)) OR flourish* OR Holistic health OR Thrive OR thriving OR Well-being OR well being OR wellbeing OR illness OR discomfort* OR discord OR Disorder* OR stress OR Isolation OR Flourish* OR satisfaction OR purpose OR virtue OR optimism OR mastery OR mental health OR physical health OR autonomy* OR self-acceptance OR self-determination OR resilience OR personal growth OR vital* OR engage* OR self-esteem OR Health status OR stability OR self-actualization OR fulfill*) AND AB (Measur* OR instrument* OR survey* OR question* OR Effect* OR associat* OR assess* OR Scale* OR Subscale* OR inventor* OR evaluat*)

Supplemental Table S2

Additional Study Characteristics Details for Studies Reporting Associations of Decisional and/or Emotional Forgiveness with Indicator/s of Well-being

Author/s	Year	Indicator/s assessed by dimension/s of well-being	Religious affiliation
Holeman et al.	2011	Psychological (intrusive event-related thoughts)<br>Spiritual (sacred loss, sacred desecration, self-rated religiosity)	Protestant (84%)<br>Catholic (2%)<br>Other religion (9%)<br>Religiously unaffiliated (5%)
Watkins et al.	2011	Volitional (conciliatory behavior)	NR
Scherer et al.	2012	Volitional (conciliatory behavior)	NR
Hook et al.	2013	Social (closeness with transgressor)<br>Volitional (conciliatory behavior)	NR
Davis et al. (Study 3)	2015	Spiritual (religious/spiritual struggles)	Identified as religious/spiritual (90%)
Kurniati et al. (Study 2)	2017	Psychological (rumination)	NR
Chi et al.	2019	Social (spousal affection, marital satisfaction, marital commitment)	NR
Cowden et al.	2019	Psychological (depression symptoms, anxiety symptoms, stress symptoms)	Christian (80%)<br>Hindu (3%)<br>Muslim (4%)<br>Other religion (6%)<br>Atheist (6%)
Choe and Davis	2020	Spiritual (religious commitment)	Religious and spiritual (46%)<br>Spiritual but not religious (31%)<br>Religious but not spiritual (10%)<br>Neither religious nor spiritual (13%)
Hong et al.	2020	Psychological (self-esteem)	NR
Major et al.	2020	Psychological (emotional well-being)	NR
Kaleta and Mróz (Study 2)	2021	Psychological (anger)	NR
Byra et al.	2022	Psychological (perceived posttraumatic growth)	NR

		Social (relationship quality with transgressor)	
Mróz and Kaleta	2022	Psychological (internet addiction)	NR
Mróz et al.	2022	Psychological (positive affect, negative affect, mental distress, rumination)	NR
Wang et al. (Study 2)	2022	Psychological (perceived stress, resilience)	NR
Wu et al.	2022	Social (relationship satisfaction)<br>Volitional (gratitude)	NR
Mróz et al.	2023	Spiritual (spiritual intelligence)	NR
Byra	2024	Psychological (psychological well-being)<br>Social (relationship quality with transgressor)	NR
Kaleta et al.	2024	Social (relationship quality with transgressor)	NR
Mróz et al.	2024	Psychological (self-compassion)<br>Spiritual (religiosity)	NR
Skalski-Bednarz et al.	2024	Psychological (mental well-being)<br>Spiritual (anger towards God, faith maturity, spiritual well-being)	Eastern Orthodox (80%)<br>Catholic (17%)<br>Protestant (3%)
He et al.	2018	Social (marital quality, marital stability)	NR
Gámiz et al.	2021	Social (closeness to transgressor)	NR
Cook et al.	2022	Physical (self-rated physical health)<br>Psychological (anxiety symptoms, depression symptoms, suffering, life satisfaction, happiness, self-rated mental health, meaning in life, sense of purpose)<br>Social (content with relationships, satisfying relationships)<br>Volitional (orientation to promote good, delayed gratification)	Christian (50%)<br>Muslim (50%)
Cornish et al.	2024	Psychological (resilience, subjective well-being, anger, rumination, esteem restoration, self-accepting beliefs, self-punitiveness)<br>Social (relationship satisfaction, positive and negative relationship qualities)	NR

		Volitional (acceptance of responsibility, amends-making, value reorientation)	
Skalski-Bednarz and Toussaint	2024	Psychological (mental well-being)<br>Spiritual (spirituality)	Catholic (76%)<br>Protestant (16%)<br>Jehovah’s Witness (8%)
Skalski-Bednarz et al.	2024	Spiritual (self-transcendent experience)	Christian (76%)<br>Religiously unaffiliated (24%)
Sandage et al.	2015	Psychological (mental health symptoms)<br>Social (experiences in close relationships)	NR
Toussaint et al.	2020	Spiritual (sacred desecration, religious commitment, spirituality)<br>Volitional (gratitude)	Hindu (89%)<br>Muslim (15%)<br>Christian (4%)<br>Jain (< 1%)

Note. NR = Not reported.
